# Gamifying motor recovery: virtual reality as a therapeutic ally in pantothenate kinase-associated neurodegeneration

**DOI:** 10.1097/MS9.0000000000004122

**Published:** 2025-10-17

**Authors:** Ali Hamza, Muammar Hassan, Muhammad Talha, Mahnoor Fatima

**Affiliations:** a Department of Medicine, Mayo Hospital, Lahore, Pakistan; b Department of Medicine, King Edward Medical University, Lahore, Pakistan; c Shaheed Ziaur Rahman Medical College, Rajshahi Medical University, Bogura, Bangladesh

*Dear Editor,*

Pantothenate kinase-associated neurodegeneration (PKAN) is a rare disorder with a severe progressive course characterized by dystonia and impaired motor function resulting from mutations of PANK2, affecting basal ganglia circuits important for movement control. Dystonia in PKAN radically impairs the quality of life, especially in children, for whom limited therapeutic options are available. In recent years, developments have indicated the future potential of new rehabilitation technologies with virtual reality (VR) to restore motor function by supporting the neuroplasticity processes^[1]^. This is in line with the TITAN Guidelines on the need for transparency in Artificial Intelligence (AI) use in health care^[2]^.

The VR-based motor training platforms offer gamified tasks that keep patients engaged in repetitive but goal-directed movements while receiving instantaneous visual and sensory feedback. Such augmented motivation and attentional engagement are potent facilitators of neuroplastic adaptation in motor rehabilitation. Several randomized controlled trials conducted in related dystonic and movement disorders have shown significant improvements in motor control and coordination with VR interventions vis-a-vis conventional therapies, thereby substantiating their clinical efficacy. The VR tasks thus allow highly controlled and customizable motor demands for personalized rehabilitation programs^[3,4]^.

Recent neurophysiological studies of patients with PKAN emphasize the disconnection of major functional connectivity within motor networks linking the basal ganglia and the motor cortices, which correlate with the severity of the disease. Motor training in a VR environment is expected to activate such neural circuits and encourage recruitment of plasticity and compensation pathways. Simultaneously, Electroencephalogram (EEG) during the VR session gives great valuable objective biomarkers for developing individualized dynamic tracking of intervention progress. Such an integration of neuroradiology and VR opens a new frontier for precise neuromodulation in PKAN dystonia^[5,6]^.

PKAN patients experience several challenges to VR platform accessibility, such as high device cost, usability, and the obligatory need for clinical supervision. To globalize therapeutic reach and improve access, there is a potential need to address these obstacles, add tele-rehabilitation methods, and provide low-cost consumer VR technology. For best utilization of neurosurgical benefits, treatment guidelines should prioritize early intervention of VR rehabilitation training as an adjunct to standard pharmacological and cerebral stimulation therapy^[7]^.

To conclude, VR-based motor training is a strong and scientifically supported complement for patients with PKAN who experience dystonia-related motor dysfunction. Multicenter trials with bigger cohorts and standardized procedures are necessary to compile these results in the future. To improve patient outcomes and increase access, it is recommended that VR rehabilitation be integrated into pediatric neurology curricula and neurodegenerative disease management policies.

## Data Availability

Not applicable to this study.

## Research registration unique identifying number (UIN)

No human subjects were involved as the type of research being submitted is a Letter to the Editor.
